# Lymphocytic Hypophysitis: Differential Diagnosis and Effects of High-Dose Pulse Steroids, Followed by Azathioprine, on the Pituitary Mass and Endocrine Abnormalities — Report of a Case and Literature Review

**DOI:** 10.1100/tsw.2010.24

**Published:** 2010-01-21

**Authors:** Lorenzo Curtò, Maria L. Torre, Oana R. Cotta, Marco Losa, Maria R. Terreni, Libero Santarpia, Francesco Trimarchi, Salvatore Cannavò

**Affiliations:** ^1^ Department of Medicine and Pharmacology, Section of Endocrinology, University of Messina, Italy; ^2^ Department of Neurosurgery, San Raffaele Scientific Institute, University Vita-Salute, Milan, Italy; ^3^ Department of Pathology, San Raffaele Scientific Institute, University Vita-Salute, Milan, Italy; ^4^ Translational Research Unit, Department of Oncology, Hospital of Prato, Istituto Toscana Tumori, Florence, Italy

**Keywords:** autoimmune hypophysitis, antipituitary antibodies, fibrosis, pituitary mass, magnetic resonance imaging

## Abstract

We report on a man with a progressively increasing pituitary mass, as demonstrated by MRI. It produced neurological and ophthalmological symptoms, and, ultimately, hypopituitarism. MRI also showed enlargement of the pituitary stalk and a dural tail phenomenon. An increased titer of antipituitary antibodies (1:16) was detected in the serum. Pituitary biopsy showed autoimmune hypophysitis (AH). Neither methylprednisolone pulse therapy nor a subsequent treatment with azathioprine were successful in recovering pituitary function, or in inducing a significant reduction of the pituitary mass after an initial, transient clinical and neuroradiological improvement. Anterior pituitary function evaluation revealed persistent hypopituitarism. AH is a relatively rare condition, particularly in males, but it represents an emerging entity in the diagnostic management of pituitary masses. This case shows that response to appropriate therapy for hypophysitis may not be very favorable and confirms that diagnostic management of nonsecreting pituitary masses can be a challenge. Clinical, imaging, and laboratory findings are useful for suggesting the diagnosis, but pituitary biopsy may be necessary to confirm it.

## INTRODUCTION

Pituitary adenomas account for about 10–15% of all intracranial neoplasms, but other benign tumors, metastases, aneurysms, and inflammatory, granulomatous, or infiltrative processes can be diagnosed in the sellar region[1,2]. A correct diagnosis is mandatory for appropriate treatment of pituitary masses; namely, when neurological and ophthalmological symptoms occur. The autoimmune hypophysitis (AH) clinical course is uncertain, and occasional, spontaneous resolution or improvement of function and regression of pituitary mass have been described[3,4,5,6,7,8,9]. For this reason, an accurate follow-up is required in subclinical patients, while the treatment must be individualized in clinically symptomatic patients. We report the history of a man with a pituitary mass extending into the left cavernous sinus, which showed progressive growth over a few months. It induced progressive neurological and ophthalmological symptoms, and hypopituitarism. On the basis of clinical course, laboratory, and MRI features, AH was suspected before surgery, but a biopsy was performed to exclude a primary pituitary lymphoma (PL). In our case, neither methylprednisolone pulse therapy nor azathioprine treatment was successful in recovering pituitary function or in reducing the mass after a transient, clinical, and neuroradiological improvement.

## CASE REPORT

A 38-year-old man complained of sudden diplopia with subacute left ophthalmoplegia, blurred vision, and subcontinous headache. Neuro-ophthalmological evaluation revealed left oculomotor nerve palsy (N III), but normal visual acuity. Visual field analysis was normal. MRI showed enlarged and hyperintense adenohypophysis, with a small quantity of relatively hypointense pathological tissue in the left cavernous sinus. The pituitary stalk was thickened, but not deviated. Gd-DTPA administration showed intense, homogeneous enhancement of the pituitary mass and of the contiguous dural plane. The third, fourth, and sixth cranial nerves inside the cavernous sinus appeared hypointense (Fig. 1). The patient did not complain of symptoms of hypo- or hyperpituitarism, nor of diabetes insipidus. Physical (BMI 27 kg/m$^2$, blood pressure 130/80 mmHg, and pulse rate 84/min) and neurological examinations were unremarkable. Serum PRL levels were slightly increased (45 µg/l), but the remaining pituitary function was normal. Hematological parameters were within the normal range. Diplopia, left ophthalmoplegia, and headache improved spontaneously during diagnostic management. For this reason, treatment was limited to the normalization of serum PRL levels by cabergoline administration (0.25 mg/week p.o.). Four months later, the patient complained of weakness, nausea, headache, erectile dysfunction, and severe retro-orbital pain. Body weight had increased (BMI 30 kg/m$^2$) and blood pressure was low (90/50 mmHg). A routine blood test revealed elevated inflammation parameters (ESR 24 mm/h, C-reactive protein 7.5 mg/l, fibrinogen 447 mg/dl), but was otherwise normal. Endocrine evaluation demonstrated panhypopituitarism (Table 1). Detection of antipituitary antibodies (APA) by indirect immunofluorescence assay showed an increased titer of APA (titer = 1:16).

Moreover, the evaluation of the HLA system showed DR2, DR3, and DR52 as predominant class II haplotypes. Abdominal ultrasonography and chest X-ray were normal. A new pituitary MRI showed a mild increase of the left-side intracavernosal part of the mass, whereas other findings were unchanged (Fig. 2). Replacement therapy with L-thyroxine (75 µg/day p.o.), cortisone acetate (25 mg/day p.o.), and testosterone enantate (250 mg/28 days, i.m.) was started. As the tumor was growing in the cavernous sinus, a pituitary biopsy was performed. It showed extensive destruction of anterior pituitary acini, with lymphocytes and plasma cells infiltrating the parenchyma of the adenohypophysis. Immunohistochemistry demonstrated inflammatory infiltrate stained with lymphoid CD20-B and CD3-T cell markers, suggesting the diagnosis of AH (Fig. 3). Coexistence of other autoimmune diseases was excluded on the basis of accurate history, physical examination, and routine laboratory tests.

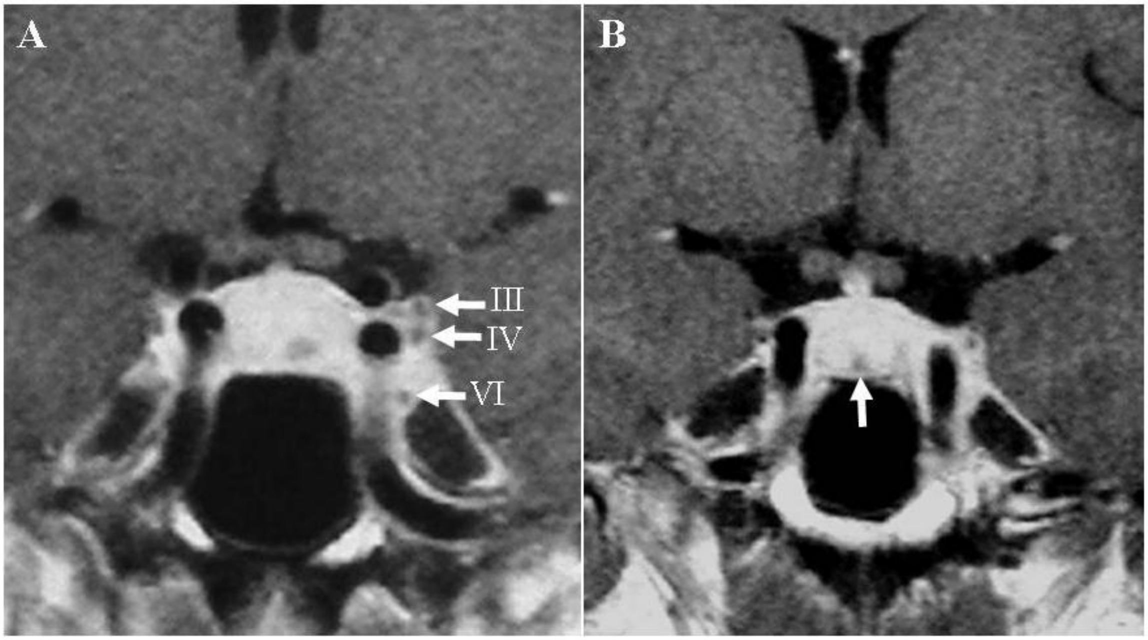

**FIGURE 1.** Coronal FSE T1-weighted imaging after Gd-DTPA administration MRI of the head. (A) Strong enhancement of the inflammatory tissue inside hypophysis and left cavernous sinus. The third, fourth, and sixth cranial nerves inside cavernous sinus (arrows) appear hypointense. (B) The pituitary focus also appears relatively hypointense (arrow).

**Table 1.** Clinical presentation, baseline and appropriately stimulated hormone values and inflammation parameters of the patient with autoimmune hypophysitis at presentation, during replacement therapy and after methylprednisolone pulse therapy and azathioprine treatment.

| | Basal evaluation | 1st control | 2nd control | 3rd control | Methylprednisolone pulse therapy | | Azathioprine |
|---|---|---|---|---|---|---|---|
| CLINICAL PRESENTATION | Headache, diplopia, ophthalmoplegia | Asthenia, nausea, erectile dysfunction. Weigth increase, hypotension. Left retro-orbital pain, headache. | | | Improvement of neurological and endocrine symptoms | | |
| TREATMENT | Cabergoline 0.25 mg/week, p.os | | L-T4 75 µg/day Cortone acetate 25 mg/day Testosterone enantate 250 mg every 28 days | TNS[&] (BIOPSY) | Testosterone enantate 250 mg every 28 days | | L-T4 75 µg/day Cortone acetate 25 mg/day Testosterone enantate 250 mg every 28 days |
| PRL µg/L (5-25) | 45.2 | <2 | | | 14.3 | | 19.3 |
| GH peak* ng/ml | 9.8 | **8.3** | | | **5.8** | | **5.1** |
| IGF-1 SD | -0.9 | -1.0 | -1.0 | | | | **-2.0** |
| FSH / LH U/L | 2.5 / 1.5 | 1.5 / 0.4 | 2.5 / 2.5 | 4.0 / 3.0 | 3.9 / 9.9 | | **0.6 / 0.3** |
| Testosterone ng/dl | **164** | **20** | **22** | 381 | 688 | | **54** |
| TSH (peak^) mU/L | 0.7 (4.7) | **0.2 (0.9)** | | | 1.2 (5.2) | **0.12** | **0.05** |
| Free T4 pmol/L | 13.3 | **10.3** | | | 19.2 | **8.4** | **10.7** |
| Cortisol (peak§) ng/ml | 150 (300) | **<10 (17)** | | | 123 (254) | **10** | **10** |
| ESR[#] mm/h | | **24** | **45** | **35** | 7 | | |
| C-reactive protein mg/L | | **7.5** | **10.0** | **12.2** | 0.4 | | |
| Fibrinogen mg/dl | | **447** | **512** | **492** | 256 | | |

Dynamic evaluation of pituitary function was performed by the i.v. administration of thyrotropin-releasing hormone (^TRH 200 µg i.v.), corticotropin-releasing hormone (§hCRH 100 µg i.v.) and *Arginine (0.5 g/kg) plus GHRH (1µg/kg) i.v. The peaks of hormone levels and the normal values of serum PRL concentrations are in parentheses. Low hormone levels and high inflammation parameters are highlighted in boldface. [&]TNS: transsphenoidal; [#]ESR: erythrocyte sedimentation rate.

## Response to Corticosteroid Therapy

After pituitary biopsy, high-dose methylprednisolone pulse therapy (HDMPT: 1000 mg/day i.v. over 30 min for 3 days consecutively during the first 2 weeks, 500 mg/day over 30 min for 3 days consecutively during the third week, and 500 mg/day over 30 min on alternate days for three times during the last 2 weeks) was started. This treatment was repeated 1 month later. HDMPT was well tolerated and the patient reported an improvement in neurological and endocrine symptoms so that, after the first cycle of

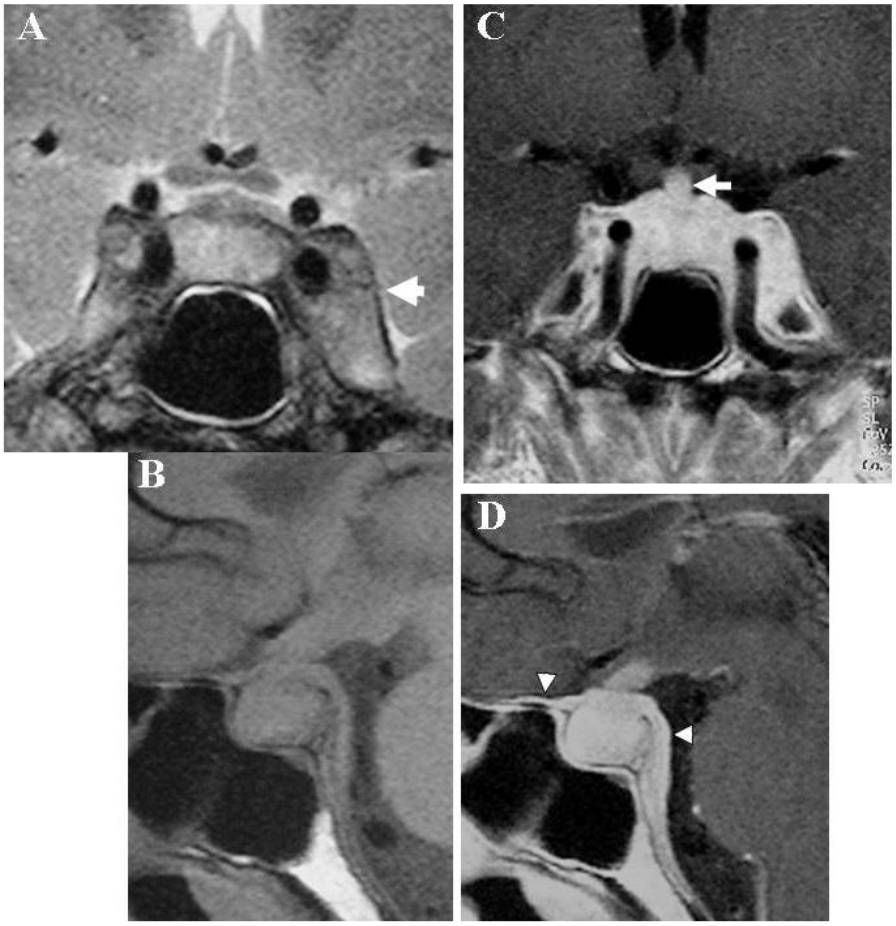

**FIGURE 2**. (A) Coronal FSE T2-weighted image MRI demonstrating mild increase of intracavernous pathological tissue (arrow). (B) Sagittal FSE T1-weighted image MRI showing cancellation of normal high signal of the posterior lobe of hypophysis. The neurohypophysis is normally hyperintense on T1-weighted images because of phospholipidic components of oxytocin and vasopressin. (C) Coronal FSE T1-weighted image after Gd-DTPA administration MRI shows persistence of infundibular stalk thickening (arrow). (D) Sagittal FSE T1-weighted image after Gd-DTPA administration MRI demonstrates a persistent thickening of the dura at clivus and at sphenoidal plane (arrow heads).

methylprednisolone, L-thyroxine and cortisone acetate, but not testosterone enantate, administration was withdrawn. Two months later, clinical improvement was observed and another neuroradiological evaluation showed a marked shrinkage of the mass. Pituitary function was partially recovered. However, 3 months after the end of the second cycle of HDMPT, the patient complained of frontal headache, asthenia, hypotension, fever (38°C), nausea, and vomiting. Pituitary MRI was unchanged, but endocrine investigation demonstrated the recurrence of secondary hypothyroidism and hypoadrenalism. After a third cycle of HDMPT, pituitary function had not been recovered, and replacement therapy with L-thyroxine and cortisone acetate was started again (Table 1). Unmasked diabetes insipidus was excluded.

## Response to Azathioprine Treatment

When endocrine function worsened again and a further MRI showed a mild regrowth of the pituitary mass, treatment with azathioprine was started at a dose of 150 mg/day. However, the drug dosage was titrated to 100 mg/day over the second month because of increased serum γ-glutamil transferase (γ-GT)

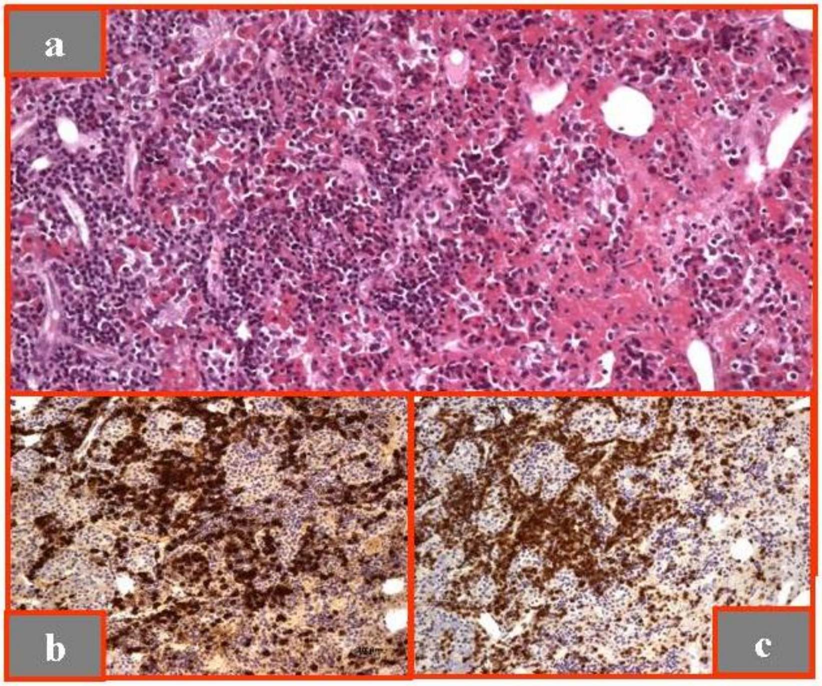

**FIGURE 3.** Histopathological detection showing extensive destruction of anterior pituitary acini with lymphoplasmacytic infiltration. (a) Lymphocytes and plasma cells infiltrate the parenchyma of the adenohypophysis (hematoxylin-eosin stain); (b) inflammatory infiltrate stained with lymphoid CD20-B cell marker; (c) inflammatory infiltrate stained with lymphoid CD3-T cell marker.

levels. After 3 months of azathioprine, a new MRI revealed inflammatory tissue with a strong enhancement visible inside adenohypophysis and left cavernous sinus, enlargement of the gland, and a "dural tail" sign still present (Fig. 4). Endocrine re-evaluation showed persistent abnormality of the pituitary function, with normalization of PRL levels (Table 1).

## DISCUSSION

AH is a chronic inflammatory disease that affects the pituitary gland and can induce neurological, ophthalmological, and endocrine damage. It was first described by Goudie and Pinkerton in 1962[10] and was considered a rare disorder until a few years ago. In recent years, it has been reported more frequently. This might be the result of improved methodologies for recognizing the disorder, instead of a true increase in incidence. Typical clinical features of AH are female preponderance, presentation during pregnancy or in the postpartum period, and association with other endocrine autoimmune disorders[10,11,12,13,14,15].

In our patient, MRI showed typical radiological signs of hypophysitis. However, enhancement (both before and after Gd-DTPA) was inhomogeneous and the typical "tent-shape" appearance was absent. Moreover, extrasellar expansion was asymmetrical with infiltration of the left cavernous sinus. Normal hyperintensity of the neurohypophysis on T1-weighted precontrastographic images was also absent, but the patient was not diagnosed with diabetes insipidus.

The detection of high titers of APA in the serum of patients can also be considered as another marker of AH. In our case, the high titers of APA, evaluated only before pituitary biopsy, could support the suspect of this disease. However, many studies have demonstrated that APA specificity is low, as they have also been detected in patients with idiopathic hypopituitarism, pituitary tumors, or autoimmune

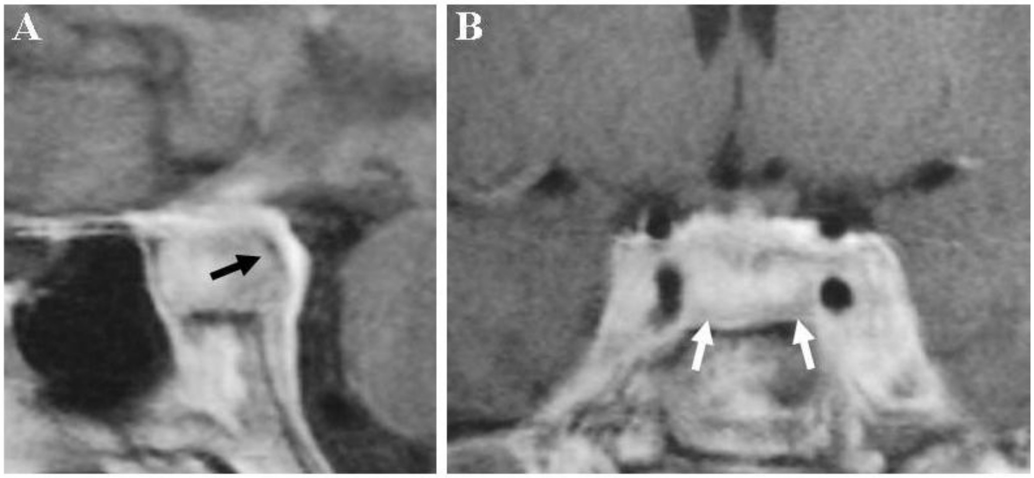

**FIGURE 4.** MRI evaluation after azathioprine treatment. (A) Sagittal FSE T1-weighted image after Gd-DTPA administration shows inflammatory tissue with a strong enhancement still visible inside adenohypophysis and left cavernous sinus. The gland is enlarged. The dural tail sign is still present (black arrow). (B) Coronal FSE T1-weighted image after Gd-DTPA administration demonstrating the hypointense rim, possibly fibrotic, also visible inside the parenchyma (white arrows).

nonpituitary diseases[11,14,16]. Personal or familial occurrence of other autoimmune diseases was not reported in our case, but HLA evaluation showed that the predominant class II molecules were DR2, DR3, and DR52, haplotypes frequently observed in patients affected by different autoimmune diseases. It has been proposed that HLA-DR genes may be closely related, in some subjects, with the genes directly responsible for the genesis of the autoimmune response[17]. In fact, the local aberrant expression of HLA-DR antigens by epithelial cells, and their subsequent capacity to present surface autoantigens to T lymphocytes, can act as a possible mechanism in the pathogenesis of endocrine autoimmunity. In our patient, the tumor MRI characteristics, its rapid growth and lateral extrasellar extension, and the occurrence of neurological and endocrine abnormalities could also be in accordance with the diagnosis of PL. Secondary pituitary localization was reported in about a quarter of patients with systemic or central nervous system (CNS) lymphomas, while primary involvement of the pituitary gland was only demonstrated in a few cases. In our patient, systemic or CNS lymphoma could be excluded on the basis of laboratory and imaging (abdomen ultrasonography and chest X-ray) findings, but not PL. The male-to-female ratio of PL is 2:1 with a peak incidence around the age of 60 years[12], while the ratio ranges from 1:5[18] to 1:8[19] in AH which, on the contrary, shows a predilection for young women[11,20]. Previous studies demonstrated that cranial nerve involvement affected about 40% of patients with PL[12], while it was rare in those with AH[21]. On the contrary, the incidence of hyperprolactinemia, isolated or multiple hypopituitarism, and diabetes insipidus was very similar in both diseases[12]. Moreover, Giustina et al.[12] hypothesized that AH could represent a potential risk factor for PL development[22], by analogy with other autoimmune endocrine diseases such as Hashimoto's thyroditis[23]. Surgical biopsy is useful to clarify the differential diagnosis. Management can be limited to strict surveillance in patients with AH, when pituitary size increases slightly, neurological symptoms are absent, and endocrine function is intact, or when clinical improvement occurs spontaneously. On the contrary, treatment is mandatory in cases in which a dramatic increase of pituitary size, neurological and neuro-ophtalmic involvement, or hypopituitarism occur. Neurosurgery is not considered a first-line treatment in either AH or PL, but debulking of the pituitary mass can improve neurological and neuro-ophthalmic symptoms in both cases. In patients with PL, radiotherapy is considered the first-line therapy[12], while chemotherapy can be used, both alone[24] when the former treatment is not effective and in addition to radiotherapy[25,26]. However, prognosis of this disease is poor, with survival <6 months in about 30% of cases[12]. As with all malignant tumors, a better outcome is related to early diagnosis. In patients with AH, steroids are the treatment of choice, despite the fact that some studies have demonstrated that this therapy cured the disease in no more than 30% of cases and that its long-term effectiveness remains questionable[27,28,29]. It is noteworthy that the outcome of AH is based on the extension of fibrosis into the pituitary gland, which frequently causes the failure of steroid treatment and the irreversible impairment of endocrine function. On the other hand, steroids improved the clinical course of the disease transiently in some patients with PL. Glucocorticoid pulse therapy and prolonged low-dose corticosteroid administration have been proposed as first-choice conservative treatments, capable of shrinking the pituitary mass and recovering pituitary function[27,28,29,30]. A surgical approach is preferred in the presence of intracranial brain and/or chiasm compression[31], ineffectiveness of corticosteroid therapy, and impossibility of establishing a diagnosis of AH by noninvasive diagnostic criteria[20,32,33]. Unfortunately, in our patient, in accordance with other cases reported in the literature[29,32], high-dose methylprednisolone pulse therapy was only transiently effective, and was followed by the re-growth of the mass and a new impairment of pituitary function. Recently, other anti-inflammatory and immunosuppressive drugs, such as methotrexate[34], cyclosporine-A[35], or azathioprine[36], have been proposed as alternative medical treatment. However, their long-term efficacy must be verified[11]. In our patient, 3 months' therapy with azathioprine was not successful in recovering pituitary function or in inducing a significant reduction of the pituitary mass after an initial, transient clinical and neuroradiological improvement. High levels of PRL have been described in the active phase both of some nonorgan-specific and organ-specific autoimmune diseases, and also in patients with AH, and a possible role of antiprolactinemic drugs in inactivating the immune process has been hypothesized. The increased PRL levels could be secondary to the inflammatory process of the pituitary gland, but they might also play a role in increasing the activity of the immune process. For this reason, APA detection in patients with idiopathic hyperprolactinemia should suggest the presence of a silent AH[37]. Furthermore, AH has been, recently, reported as a serious immune-related side effect in subjects undergoing treatment with ipilimumab, a new immunotherapeutic antineoplastic agent used in the treatment of metastatic melanoma and renal cell carcinoma[38,39].

In conclusion, the case reported here confirms that the diagnostic management of nonsecreting pituitary masses can be a challenge, and that clinical, imaging, and laboratory findings are useful to suggest the diagnosis. In our patient, AH was suspected on the basis of MRI and laboratory findings, and the diagnosis was confirmed by pituitary biopsy. MRI is a useful tool in suspected cases of the disease, even if, currently, there is no evidence that it is able to diagnose the extension of fibrosis into the pituitary gland properly. This can be a critical point in predicting the outcome of the disease after medical treatment, as fibrosis reduces the chances of recovery from hypopituitarism.
